# The proteostasis paradox: from systemic collapse in aging to pathway-specific addiction in prostate cancer

**DOI:** 10.3389/fcell.2026.1755668

**Published:** 2026-01-15

**Authors:** Danhong Guo, Yaoyao Peng, Yanlan Yu

**Affiliations:** 1 Nursing Department, Sir Run Run Shaw Hospital, Zhejiang University School of Medicine, Hangzhou, China; 2 Urology Department, Sir Run Run Run Shaw Hospital, Zhejiang University School of Medicine, Hangzhou, China

**Keywords:** androgen receptor signaling, cellular senescence, prostate cancer progression, proteostasis dysregulation, therapeutic targeting

## Abstract

Aging is the primary risk factor for prostate cancer (PCa), characterized biologically by a systemic collapse of proteostasis networks. Paradoxically, rather than succumbing to this decline, PCa cells exploit it, developing a “proteostasis addiction” to cope with persistent intrinsic stress. This review elucidates this paradox through three conceptual frameworks: the dynamic transition from age-related functional decay to tumorigenic hijacking; the specificity of oncogenic protein regulation; and the functional dichotomy (or “double-edged sword”) of proteostatic components in tumor suppression *versus* promotion. We examine how declining molecular chaperone networks are co-opted to selectively stabilize the androgen receptor (AR) and its variants. Furthermore, we explore how the ubiquitin–proteasome system (UPS) is re-engineered *via* E3 ligases and deubiquitinases (DUBs) to orchestrate the precise turnover of tumor suppressors and oncoproteins. Special attention is given to chaperone-mediated autophagy (CMA), which undergoes a reversal from age-associated suppression to hyperactivation in advanced PCa, thereby fueling metabolic adaptation and therapy resistance. Beyond the intracellular context, we discuss how proteostatic imbalances drive the senescence-associated secretory phenotype (SASP) to remodel the tumor microenvironment. Finally, we assess emerging therapeutic strategies, arguing that precision modulation of specific proteostasis nodes—such as distinct E3/DUBs or CMA pathways—represents a promising frontier to overcome castration-resistant prostate cancer (CRPC).

## 1 Introduction

Prostate cancer (PCa) is one of the most prevalent malignancies of the male genitourinary system worldwide, with both incidence and mortality showing a strong positive correlation with age, classifying it as a quintessential age-related disease (Kumar et al., 2021; Ottens et al., 2021; Kang and Jeon, 2021). Epidemiological evidence indicates that the risk of developing PCa increases sharply after the age of 40, and elderly patients often present with more aggressive clinicopathological features and faster disease progression, highlighting aging as a major risk factor for PCa (Kumar et al., 2021; Kang and Jeon, 2021; Elkenani et al., 2022). In rapidly aging countries such as China, demographic shifts have contributed to a continuous rise in both the incidence and detection rate of PCa (Zhang et al., 2021). Beyond chronological age, metabolic factors commonly observed in older populations, including obesity and altered dietary patterns, have also been implicated in elevating the risk of PCa (Ottens et al., 2021). Importantly, advanced age is associated not only with more aggressive disease characteristics but also with shorter overall survival and higher mortality (Kumar et al., 2021; Elkenani et al., 2022).

At the molecular level, a hallmark of aging is the systemic decline in proteostasis, the cell’s capacity to maintain protein homeostasis. Proteostasis relies on three interconnected pillars: proper protein folding mediated by molecular chaperones, precise degradation of proteins *via* the ubiquitin–proteasome system (UPS), and autophagy pathways, all of which collectively safeguard protein quality and ensure cellular functionality (Kumar et al., 2021; Ottens et al., 2021; Liang et al., 2023). With age, these mechanisms progressively lose efficiency, leading to the accumulation of misfolded proteins and damaged organelles, ultimately precipitating cellular dysfunction and aging-related phenotypes (Selvaraj et al., 2025; Sharma et al., 2025). Paradoxically, in cancer—and particularly in PCa—tumor cells do not passively succumb to this decline. Instead, they actively “hijack” and remodel the deteriorating proteostasis network, transforming it into a vital machinery that supports malignant phenotypes and enables adaptation to intrinsic and extrinsic stressors (Liang et al., 2023). Thus, aging, proteostasis imbalance, and PCa form a perilous triad: aging provides a permissive environment, while proteostasis dysregulation offers the molecular scaffold driving tumor initiation and progression.

The remodeling of proteostasis in PCa reflects a dynamic and highly selective adaptation. Whereas normal aging is characterized by decreased expression of molecular chaperones, reduced proteasome activity, and diminished autophagic flux (Ottens et al., 2021; Elkenani et al., 2022; Cóppola-Segovia and Reggiori, 2024), prostate cancer cells paradoxically reverse these trends in a context-specific manner. They selectively upregulate particular chaperones, activate key components of the UPS, or enhance chaperone-mediated autophagy (CMA), thereby converting a general loss of proteostasis into a malignancy-specific dependency. This remodeling is substrate-specific: chaperones such as HSP90 and HSP70 preferentially stabilize critical drivers like the androgen receptor (AR) and its splice variants, while E3 ligases and deubiquitinases (DUBs) within the UPS precisely regulate the degradation and stabilization of tumor suppressors and oncogenic proteins. Autophagic pathways further contribute by selectively targeting metabolic proteins, reshaping cellular metabolism to meet the demands of tumor growth (Kang and Jeon, 2021; Zhang et al., 2021; Chakraborty and Edkins, 2023). This highly specific “client protein network” not only underlies tumor survival but also represents a promising target for precision therapeutics.

Proteostasis pathways in PCa also exhibit a double-edged sword effect, functioning both as tumor suppressors and promoters depending on the context. For example, generalized UPS decline may stabilize tumor suppressors such as p53 and exert anti-tumor effects, yet tumor cells can exploit select UPS components to degrade these suppressors selectively (Kubbutat et al., 1997). Similarly, autophagy can prevent tumor initiation by clearing damaged organelles, while later supporting tumor growth by recycling macromolecules to provide energy and biosynthetic precursors (Liang et al., 2023; Selvaraj et al., 2025). This duality emphasizes that therapeutic strategies targeting the proteostasis network must carefully balance tumor-suppressive and tumor-promoting effects to achieve optimal intervention.

To elucidate the paradox described herein, we explicitly define “Systemic Decline” as the age-related, global loss of protein quality control capacity, which creates a permissive environment for genomic instability. In contrast, “Pathway-Specific Addiction” refers to the tumor-specific upregulation and hijacking of select proteostasis nodes (such as specific chaperones or CMA pathways) to cope with the enhanced proteotoxic stress of malignancy.

Collectively, aging-driven proteostasis decline is not a simple unidirectional degenerative process but a dynamic and exploitable biological phenomenon. Understanding its intertwined dimensions of dynamic transition, substrate specificity, and context-dependent duality provides critical insights into the pathogenesis of prostate cancer and informs the development of novel therapeutic strategies.

## 2 Molecular chaperone networks: from cellular guardians to tumor “accomplices”

### 2.1 Functional decline in aging

Aging is a systemic, multidimensional biological process, one hallmark of which is the disruption of proteostasis. Numerous studies have demonstrated a marked decline in the heat shock response (HSR) during aging (Llewellyn et al., 2024; Llewellyn et al., 2023). The HSR, primarily regulated by the transcription factor HSF1, is activated in response to heat shock or other stressors, inducing the expression of molecular chaperones such as HSP90 and HSP70 to correct misfolded proteins (Hu et al., 2020; Gu et al., 2025; Kose et al., 2022; Smadja and Abreu, 2025; Schroeder et al., 2024). However, in aged individuals, this mechanism progressively fails, resulting in reduced chaperone expression and impaired function (Llewellyn et al., 2024).

Molecular chaperones are critical regulators of intracellular proteostasis, facilitating proper protein folding, assembly, and degradation (Hu et al., 2020; Gu et al., 2025; Kose et al., 2022). Among these, HSP90 and HSP70 are particularly essential, as they not only directly assist client protein folding but also coordinate with protein degradation systems, including the ubiquitin–proteasome pathway, to prevent the accumulation of misfolded proteins (Llewellyn et al., 2023; Kinger et al., 2023). With advancing age, both the expression and functionality of HSP90 and HSP70 decline (Llewellyn et al., 2024), leading to exacerbated protein aggregation and contributing to the onset of protein-folding-related disorders (Llewellyn et al., 2024). Additionally, imbalances in protein synthesis and turnover (Llewellyn et al., 2024; Llewellyn et al., 2023). Further compromise the cell’s capacity to restore homeostasis through the chaperone network, accelerating protein damage and the progression of aging-related pathologies.

### 2.2 Dynamic remodeling and “chaperone addiction” in prostate cancer

In contrast to the decline observed in aging, tumor cells subjected to persistent intrinsic stress—such as genetic mutations and metabolic perturbations—develop an “addiction” to chaperone proteins, a phenomenon commonly referred to as non-oncogene addiction. This dependency is particularly pronounced in prostate cancer (Arneaud and Douglas, 2016; Powers and Workman, 2006).

In castration-resistant prostate cancer (CRPC), HSP90 and HSP70 family chaperones are significantly upregulated, maintaining the stability of the androgen receptor (AR) and its splice variants, thereby supporting cancer cell survival in androgen-depleted environments (Ni et al., 2010; Moses et al., 2018). HSP90 forms complexes with co-chaperones, including FKBP51 and p23, to enhance AR stability and transcriptional activity (Ni et al., 2010). Meanwhile, the HSP70/HSP40 axis plays a crucial role in sustaining AR variant stability, and inhibition of this axis accelerates AR degradation (Moses et al., 2018).

Molecular chaperones also facilitate dynamic remodeling of transcriptional complexes, promoting cancer cell lineage plasticity (Morimoto, 2002). Moreover, these chaperone networks support lineage plasticity and the development of Neuroendocrine Prostate Cancer (NEPC), allowing tumor cells to transdifferentiate and evade AR-targeted therapies. For instance, in prostate cancers with TP53 and RB1 loss, upregulation of SOX2 can drive a transition from AR-dependent to neuroendocrine phenotypes, enabling evasion of therapeutic pressure (Mu et al., 2017; Ku et al., 2017). Mitochondrial chaperone TRAP-1, a mitochondrial homolog of HSP90, is highly expressed in prostate cancer and contributes to tumor progression by suppressing apoptosis (Leav et al., 2010). Clinical genomic analyses have further elucidated the complex molecular landscapes of CRPC and neuroendocrine prostate cancer (NEPC) (Robinson et al., 2015; Beltran et al., 2011; Beltran et al., 2016; Dardenne et al., 2016), highlighting chaperone-dependent mechanisms as key drivers of tumor evolution and therapeutic resistance.

### 2.3 Specificity in client protein networks centered on the androgen receptor

The androgen receptor (AR) and its splice variants, such as AR-V7, are central mediators of CRPC progression and therapeutic resistance (Zheng et al., 2022). HSP90 maintains the stability of the AR ligand-binding domain (LBD) through its chaperone cycle (Gillis et al., 2013; Li et al., 2013; Guo et al., 2009; Dehm et al., 2008; Hu et al., 2012; Ratajczak et al., 2022). Although AR-V7 lacks the LBD, its stability remains partially dependent on HSP90 (Gillis et al., 2013; Shafi et al., 2013). Studies have shown that HSP90 inhibitors, such as geldanamycin and its derivatives, effectively degrade full-length AR (AR-FL) but exhibit variable effects on AR-V7, partly by modulating its mRNA splicing (Shafi et al., 2013; Ferraldeschi et al., 2016).

HSP70, in contrast, plays a more direct and critical role in AR-V7 stability. HSP70, together with HSP40, facilitates AR folding and inhibits E3 ligase STUB1-mediated degradation, thereby maintaining AR-V7 protein levels (Liu et al., 2018; Xu et al., 2023). HSP70 inhibition disrupts its interaction with AR-V7, promotes nuclear translocation of STUB1, and induces AR-V7 degradation, restoring sensitivity to enzalutamide (Liu et al., 2018; Xu et al., 2023; Kita et al., 2017). Clinically, AR-V7–positive patients are generally resistant to abiraterone and enzalutamide, correlating with poor prognosis (Antonarakis et al., 2014; Antonarakis et al., 2017; Hu et al., 2009).

Therapeutically, HSP90 inhibitors such as Onalespib and Bruceantin have been employed to target the AR/AR-V7 axis (Ferraldeschi et al., 2016; Moon et al., 2021). Despite early clinical limitations related to toxicity and efficacy (Hu et al., 2012), combination therapies and novel inhibitors demonstrate promise. HSP70 inhibitors, including JG98 and Ver155008, show preclinical efficacy by synergistically enhancing AR-V7 degradation and drug sensitivity (Xu et al., 2023; Kita et al., 2017). Additionally, PROTAC-based bifunctional peptides (Ma et al., 2024) and novel compounds targeting AR-V7 transcriptional regulation (Dehm et al., 2008; Liu C. et al., 2021; Bradley, 2019) are under active investigation.

In summary, the molecular chaperone network exhibits a bipolar role in aging and tumorigenesis: functional decline during aging leads to protein aggregation, whereas in tumors, chaperones are “hijacked” to sustain malignant phenotypes. In prostate cancer, the specific regulation of AR/AR-V7 by HSP90 and HSP70 exemplifies tumor cell chaperone dependency, providing a strong theoretical foundation for the development of innovative therapeutic strategies.

## 3 The ubiquitin–proteasome system (UPS): a precision-regulated “double-edged sword”

The ubiquitin–proteasome system (UPS) is a central intracellular protein degradation pathway that plays a critical role in maintaining proteostasis, thereby regulating cellular function, aging, and disease development (Finley, 2009; Peng et al., 2022). By tagging target proteins with ubiquitin and subsequently degrading them *via* the proteasome, the UPS maintains dynamic intracellular protein homeostasis. However, its function undergoes significant alterations under pathological conditions such as aging and cancer. Notably, in prostate cancer, the UPS exhibits a “double-edged sword” effect: it can suppress early tumor development through stabilization of tumor suppressors, yet can be hijacked by cancer cells to promote tumor progression and therapy resistance (Peng et al., 2022; Chen X. et al., 2021). This section discusses the decline of UPS activity during aging and its dual role in prostate cancer, with a focus on the “targeted redirection” mechanisms mediated by E3 ubiquitin ligases and deubiquitinases (DUBs) that drive tumor progression.

### 3.1 Decline of UPS activity during aging

Proteasome activity generally declines during aging, leading to impaired clearance of ubiquitinated proteins and exacerbating proteostasis imbalance, thereby promoting aging-related pathologies (Andersson et al., 2013; Saez and Vilchez, 2014; Kelmer Sacramento et al., 2020; Giannini et al., 2013). Age-associated proteasome dysfunction arises from multiple mechanisms. First, aggregates of misfolded proteins, such as inclusion bodies, directly obstruct substrate recognition and proteolysis by the proteasome (Andersson et al., 2013; Bence et al., 2001). Second, decreased intracellular ATP levels impair assembly of the 26S proteasome, which relies on ATP-driven subunit association, reducing the amount of active proteasomes (Giannini et al., 2013; Vernace et al., 2007). Additionally, defects or functional loss of regulatory subunits, such as PA700/19S, compromise the proteasome’s capacity to recognize and unfold ubiquitinated proteins (Finley, 2009; Ferrington et al., 2005). Oxidative stress further exacerbates proteasome dysfunction by modifying critical proteasomal residues, such as cysteines, and inhibiting enzymatic activity (Ferrington et al., 2005; Shringarpure et al., 2003). Simultaneously, decline in molecular chaperone function, e.g., HSP90, limits proteasome remodeling and repair (Nanduri et al., 2015). Collectively, these alterations result in the accumulation of ubiquitinated proteins, disruption of proteostasis, and acceleration of aging-related disorders such as neurodegenerative diseases and cardiomyopathies (Saez and Vilchez, 2014). Notably, in aged brains, reduced proteasome activity is accompanied by ribosomal stoichiometric imbalance and protein aggregation, further exacerbating cellular dysfunction (Kelmer Sacramento et al., 2020).

### 3.2 The “double-edged sword” in prostate cancer

In prostate cancer, the UPS exhibits a context-dependent “double-edged sword” effect. Partial UPS dysfunction in early tumorigenesis may inhibit cancer growth, whereas in advanced tumors, cancer cells exploit UPS mechanisms to promote proliferation, invasion, and therapeutic resistance (Chen X. et al., 2021; Gouranga et al., 2023).

#### 3.2.1 Tumor-suppressive effects

During early-stage cancer, partial impairment of UPS function can stabilize tumor suppressors, exerting anti-tumor effects (Chen X. et al., 2021; Vassilev et al., 2004). For instance, UPS dysfunction may inhibit the activity of the E3 ligase MDM2, reducing p53 ubiquitination and degradation, allowing p53 accumulation to trigger apoptosis or cell cycle arrest, thereby suppressing tumor initiation (Kubbutat et al., 1997; Saez and Vilchez, 2014). MDM2 antagonists, such as Nutlin, can activate the p53 pathway by blocking MDM2–p53 interactions, inducing cancer cell apoptosis (Vassilev et al., 2004). UPS dysfunction may also stabilize other tumor suppressors, such as p27, indirectly inhibiting cellular proliferation (Halvorsen et al., 2003; Raghu et al., 2017).

#### 3.2.2 Tumor-promoting effects

In advanced prostate cancer, cancer cells selectively exploit the UPS to degrade tumor suppressors (e.g., p27, PTEN) and stabilize oncogenic proteins (e.g., c-Myc, AR), thereby promoting tumor growth and therapy resistance (Wang et al., 2007; Jichun et al., 2024). This imbalance is often driven by aberrant overexpression or mutation of E3 ligases, frequently accompanied by defects in DNA damage response (Boysen et al., 2015). For example, overexpression of the E3 ligase E6AP in prostate cancer specifically targets p27 for degradation, resulting in cell cycle dysregulation (Raghu et al., 2017). Similarly, PTEN degradation diminishes its inhibition of the PI3K/Akt pathway, enhancing cancer cell survival (Halvorsen et al., 2003). Additionally, stabilization of c-Myc by the UPS further promotes proliferation and metabolic activity (Chen X. et al., 2021; Popov et al., 2007). These mechanisms collectively drive the aggressiveness of castration-resistant prostate cancer (CRPC) and resistance to androgen deprivation therapies such as enzalutamide (Chen X. et al., 2021; Raghu et al., 2017). Inhibition of key UPS components, such as E1 or E3 enzymes, has been shown to restore tumor suppressor function and suppress tumor progression (Yang et al., 2007).

### 3.3 Specificity: targeted redirection by E3 ligases and DUBs

Prostate cancer cells hijack E3 ubiquitin ligases and deubiquitinases (DUBs) *via* “targeted redirection” mechanisms, precisely controlling the stability of key proteins to drive tumor progression and therapy resistance (Šimečková et al., 2019; Davies and Shringarpure, 2006).

#### 3.3.1 Specificity of E3 ligases

E3 ligases promote oncogenesis by selectively targeting tumor suppressors for degradation in prostate cancer (Davies and Shringarpure, 2006).

As the substrate recognition component of the SCF complex, SKP2 is overexpressed in prostate cancer, targeting tumor suppressors p27 and FOXO1 for degradation (Finley, 2009; Andersson et al., 2013; Shringarpure et al., 2003). Degradation of p27, a cell cycle inhibitor, leads to uncontrolled proliferation (Gru et al., 1997), while FOXO1, a pro-apoptotic factor, is destabilized, enhancing cancer cell survival (Soucy et al., 2009). SKP2 also promotes Twist ubiquitination, facilitating epithelial–mesenchymal transition (EMT) and stemness, accelerating CRPC metastasis (Vernace et al., 2007; Ferrington et al., 2005). Notably, SKP2 can mediate AR degradation, although this effect is context-dependent (Shringarpure et al., 2003). SKP2 inhibitors, such as Compound #25, restore p27 and FOXO1 levels and inhibit tumor growth (Finley, 2009).

Activated Akt signaling promotes MDM2-mediated phosphorylation-dependent ubiquitination and proteasomal degradation of AR, reducing its transcriptional activity and facilitating androgen-independent growth (Nanduri et al., 2015). Concurrently, MDM2 degrades p53, weakening its tumor-suppressive function (Saez and Vilchez, 2014). MDM2 antagonists, such as Nutlin, block interactions with p53 or AR, restoring tumor-suppressive activity (Saez and Vilchez, 2014). MDM2 also cooperates with other E3 ligases, such as CHIP, to further regulate AR stability (Chauhan et al., 2012).

#### 3.3.2 Specificity of DUBs

DUBs remove ubiquitin chains from oncogenic proteins, enhancing their stability and promoting prostate cancer progression and therapy resistance (Šimečková et al., 2019).

USP7 stabilizes AR by removing ubiquitin chains, promoting chromatin binding and transcription of target genes such as PSA (Kelmer Sacramento et al., 2020). In CRPC, USP7 maintains the stability of AR splice variants (e.g., AR-V7), supporting androgen-independent growth and enzalutamide resistance (Vassilev et al., 2004). USP7 also stabilizes c-Myc *via* deubiquitination, enhancing glycolysis and proliferation (Giannini et al., 2013). USP7 inhibitors, such as P5091, reduce AR and c-Myc stability, overcoming therapy resistance (Chauhan et al., 2012; Liu Z. et al., 2021).

USP14 selectively removes K48-linked ubiquitin chains from AR, prolonging its half-life and promoting cell cycle progression and proliferation (Halvorsen et al., 2003; Raghu et al., 2017). Inhibition with IU1 increases AR ubiquitination and degradation, suppressing CRPC growth and highlighting its therapeutic potential (Halvorsen et al., 2003). USP14 similarly stabilizes c-Myc and other oncogenic proteins, enhancing tumor cell survival (Giannini et al., 2013). Its activity is also linked to DNA repair and autophagy regulation, further contributing to therapy resistance (Bushweller, 2019).

#### 3.3.3 Therapeutic implications

Targeting the “targeted redirection” mechanisms of E3 ligases and DUBs has emerged as a promising strategy in prostate cancer therapy (Chen X. et al., 2021). SKP2 inhibitors, such as Compound #25, restore p27 and FOXO1 function, inducing apoptosis (Finley, 2009). MDM2 antagonists, such as Nutlin, activate the p53 pathway to inhibit tumor growth (Saez and Vilchez, 2014). USP7 inhibitors (e.g., P5091) and USP14 inhibitors (e.g., IU1) effectively reduce AR and c-Myc stability in preclinical models, overcoming enzalutamide resistance in CRPC (Vassilev et al., 2004; Halvorsen et al., 2003). Additionally, inhibitors targeting E1 or NEDD8-activating enzymes exhibit anticancer potential (Yang et al., 2007). These findings collectively indicate that precise modulation of critical UPS nodes represents a key avenue for future prostate cancer therapy (Šimečková et al., 2019).

## 4 Autophagy pathways: the cellular decision between survival and death

Autophagy is a conserved intracellular mechanism that plays a central role in maintaining cellular homeostasis, responding to stress, and regulating cell fate. Its activity exhibits marked dynamism and a “double-edged sword” effect in aging and cancer contexts (Cuervo, 2008; Cassidy and Narita, 2022; Ghosh et al., 2023). Autophagy encompasses multiple forms, including macroautophagy and chaperone-mediated autophagy (CMA). Macroautophagy involves autophagosome formation and lysosomal fusion for the non-selective degradation of cellular components, whereas CMA selectively degrades substrates containing KFERQ-like motifs through recognition by molecular chaperones (Cuervo and Dice, 2000; Loeffler, 2019; Yao and Shen, 2020). The functionality of these pathways changes with age and disease progression, declining during aging but exhibiting context-dependent reversal in prostate cancer, thereby influencing tumor initiation, progression, and therapeutic response (Cassidy and Narita, 2022; Ghosh et al., 2023).

### 4.1 Functional decline during aging

Both macroautophagy and CMA flux significantly decrease with age, resulting in impaired clearance of damaged organelles, protein aggregates, and metabolic waste, accelerating cellular functional decline and age-related pathologies (Cuervo, 2008; Cassidy and Narita, 2022; Ghosh et al., 2023; Cuervo and Dice, 2000). The reduction in macroautophagy stems from downregulation of autophagy-related genes (e.g., Beclin1 and the Atg family) and lysosomal dysfunction, which compromise the removal of damaged mitochondria and oxidized proteins (Hara et al., 2006; Komatsu et al., 2006). For example, heterozygous Beclin1 knockout mice exhibit defective autophagy that promotes tumorigenesis while accelerating neurodegenerative changes (Hara et al., 2006; Komatsu et al., 2006; Qu et al., 2003). The decline in CMA is particularly pronounced; the stability of its key receptor, LAMP-2A, on the lysosomal membrane decreases with age, weakening its complex formation with the chaperone HSPA8, and reducing substrate binding and translocation efficiency. This results in metabolic waste accumulation and impaired cellular maintenance (Cuervo and Dice, 2000; Zhang and Cuervo, 2008). Restoration of CMA activity, such as *via* LAMP-2A overexpression, improves cellular function in aged livers, highlighting its potential in anti-aging interventions (Zhang and Cuervo, 2008). Collectively, the comprehensive decline in autophagic function is a major driver of aging-related pathologies, including neurodegeneration and metabolic disorders (Cuervo, 2008; Cassidy and Narita, 2022; Ghosh et al., 2023).

### 4.2 “Dynamic reversal” and the double-edged sword effect in prostate cancer

In prostate cancer, autophagy exhibits a highly context-dependent “dynamic reversal,” acting as a tumor suppressor during early stages but switching to a pro-survival mechanism in advanced tumors to help cancer cells cope with stress (Cassidy and Narita, 2022).

Macroautophagy plays complex, stage-specific roles. During tumor initiation, macroautophagy suppresses malignancy by clearing damaged organelles, limiting inflammation, and controlling oxidative stress; Beclin1-mediated autophagy inhibits tumor formation (Qu et al., 2003; Degenhardt et al., 2006). However, in established tumors, macroautophagy is “reversed” into a tumor-promoting mechanism, recycling amino acids, fatty acids, and other metabolic substrates to support cancer cell proliferation and survival under hypoxia and nutrient deprivation (Degenhardt et al., 2006; Manzoor et al., 2022). For instance, Ras-driven autophagy maintains oxidative metabolism and tumor growth (Guo et al., 2011). In prostate cancer, this functional switch exacerbates tumor progression and therapy resistance (Manzoor et al., 2022; Feng et al., 2019).

Chaperone-Mediated Autophagy (CMA) also undergoes a functional reversal. In contrast to the decline observed in aging, CMA is markedly activated in advanced prostate cancer cells, particularly under chemotherapy or endocrine therapy-induced stress, enabling adaptation to metabolic pressure and treatment resistance (Feng et al., 2019; Fan et al., 2021). The CMA receptor LAMP2A is highly expressed in tumor tissues, forming complexes with HSPA8 to enhance substrate degradation, allowing cancer cells to withstand androgen deprivation or chemotherapeutic stress (Yao and Shen, 2020; Qu et al., 2003; Fan et al., 2021). In castration-resistant prostate cancer (CRPC), CMA activation is regulated by acetylation of the TPD52 protein, promoting adaptive survival (Fan et al., 2021). This activation highlights CMA as a key mechanism of therapeutic resistance, synergizing with macroautophagy’s pro-survival effects to drive CRPC progression (Fan et al., 2021). Clinical studies indicate that autophagy inhibition can enhance chemotherapy (e.g., docetaxel) or endocrine therapy (e.g., enzalutamide or abiraterone) efficacy, but careful consideration of the dual effects is required (Komatsu et al., 2006; Chang et al., 2024; Bandyopadhyay et al., 2008).

### 4.3 Specificity: CMA-mediated selective degradation of key substrates

CMA selectively degrades proteins containing KFERQ-like motifs, remodeling cancer cell metabolism and cell cycle regulation to support survival (Cuervo, 2008; Cassidy and Narita, 2022; Loeffler, 2019; Yao and Shen, 2020). In glycolytic regulation, CMA targets pyruvate kinase M2 (PKM2), a rate-limiting glycolytic enzyme that often exists in a low-activity dimer form to divert intermediates toward biosynthesis, supporting cancer cell proliferation (Folkerts et al., 2019; Yang and Lu, 2013). CMA dynamically regulates PKM2 activity by degrading it, balancing active and inactive forms, thereby enhancing glycolytic flux and accumulating metabolic intermediates for biosynthetic processes (Zhang and Cuervo, 2008; Folkerts et al., 2019; Yang and Lu, 2013). Concurrently, CMA degrades cell cycle inhibitors such as p27, relieving G1/S phase arrest and promoting cell cycle progression (Loeffler, 2019; Zhang and Cuervo, 2008). In prostate cancer, PKM2 overexpression correlates with tumor progression, and its silencing suppresses proliferation and induces apoptosis (Zhang and Cuervo, 2008). These mechanisms collectively reprogram metabolism to support cancer cell survival (Ghosh et al., 2023; Loeffler, 2019).

Targeting CMA core components, such as LAMP2A, has emerged as a promising strategy for treating CRPC (Qu et al., 2003). In CRPC, elevated CMA activity maintains high glycolytic flux through degradation of metabolic enzymes like PKM2 and enhances AR activity by degrading AR inhibitory factors, sustaining proliferation even under androgen blockade (Yang and Lu, 2013). LAMP2A silencing induces apoptosis in CRPC cells and suppresses xenograft tumor growth, confirming its role in survival dependence (Qu et al., 2003). Preclinical studies show that shRNA or CMA inhibitors (e.g., QX77) block CMA, leading to oxidative stress accumulation and metabolic dysregulation (Yang and Lu, 2013). Combined with enzalutamide, CMA inhibition synergistically induces tumor regression and delays resistance (Komatsu et al., 2006). Exploiting CMA-associated metabolic vulnerabilities, development of LAMP2A-targeting small molecules or monoclonal antibodies is now a key strategy to overcome CRPC resistance (Qu et al., 2003; Wong et al., 2014).

## 5 Integrated perspective: how proteostasis imbalance cooperatively drives prostate cancer

In prostate cancer, disruption of the proteostasis network—comprising molecular chaperones, the ubiquitin-proteasome system (UPS), and autophagy pathways—cooperatively converges on the androgen receptor (AR) as a central hub, driving tumor initiation, progression, and therapeutic resistance (Kim et al., 2013; López-Otín et al., 2013; Takayama and Inoue, 2013). This dysregulation extends beyond intracellular homeostasis to influence the tissue microenvironment, remodeling tumor ecology through the senescence-associated secretory phenotype (SASP), thereby promoting immunosuppression and malignant evolution (Melia et al., 2022; Krtolica et al., 2001; Acosta et al., 2008). This section integrates interactions among these three systems and explores how intracellular mechanisms impact microenvironmental dynamics.

### 5.1 Crosstalk within the proteostasis network: AR-centric regulation

Molecular chaperones, UPS, and autophagy coordinately regulate the lifecycle of AR—from synthesis and folding to activation and degradation—forming a dynamic equilibrium network. Dysregulation of this network in prostate cancer leads to aberrant AR signaling and promotes tumor progression (Kim et al., 2013; Takayama and Inoue, 2013; Shkedi et al., 2022; Lin et al., 2002).

The molecular chaperone system is critical for initial AR folding and conformational maintenance. HSP90 binds AR to maintain it in an inactive state until ligand binding triggers transcriptional activation (Kim et al., 2013; Shkedi et al., 2022; Pajonk et al., 2005); HSP70 assists in folding nascent AR polypeptides, preventing misfolding (Kim et al., 2013; Hartl et al., 2011). Overexpression of HSP60 is associated with progression to castration-resistant prostate cancer (CRPC), and its knockdown suppresses CRPC cell survival by indirectly modulating AR signaling through mitochondrial proteostasis (Shkedi et al., 2022).

UPS regulates AR stability and activity through ubiquitination and proteasomal degradation. Proteasome activity is essential for AR nuclear translocation and interaction with coactivators such as ARA70; proteasome inhibition blocks AR nuclear import, suppressing transcriptional activity (Lin et al., 2002; Frankland-Searby and Bhaumik, 2012). Ubiquitination of AR, e.g., mediated by MDM2, directly affects its degradation rate and transcriptional efficiency, and adaptive UPS alterations in CRPC (e.g., E3 ligase mutations) maintain high AR expression (Lin et al., 2002; Frankland-Searby and Bhaumik, 2012).

Autophagy is regulated transcriptionally by AR, which directly activates core autophagy genes (e.g., ATG4B, ULK1, ULK2) and lysosomal biogenesis regulator TFEB, promoting prostate cancer cell proliferation and lipid droplet catabolism to maintain energy homeostasis (Blessing et al., 2017; Kurganovs and Engedal, 2024). Under stress conditions such as androgen deprivation, autophagy compensates for UPS insufficiency by selectively degrading damaged organelles to support cancer cell survival, though excessive activation can drive therapy resistance (Blessing et al., 2017; Kurganovs and Engedal, 2024).

Tight crosstalk exists among these systems: HSP90 inhibition can induce UPS-dependent AR degradation (Shkedi et al., 2022); importantly, this relationship is causal and compensatory, as proteasome inhibition paradoxically triggers a compensatory activation of autophagy to clear ubiquitinated AR aggregates, thereby driving secondary resistance. Autophagy activation under anti-androgen therapy can clear protein aggregates not processed by UPS, sustaining AR signaling (Blessing et al., 2017; Kurganovs and Engedal, 2024); and chaperones such as HSP70 bridge UPS and autophagy, facilitating the transfer of ubiquitinated AR to autophagosomes (Kim et al., 2013; Shkedi et al., 2022). In CRPC, network adaptability is enhanced (e.g., HSP60 upregulation, autophagy gene amplification, and UPS reprogramming), allowing AR signaling to persist under low androgen conditions and even evade degradation *via* AR splice variants such as AR-V7, promoting tumor survival and metastasis (Shkedi et al., 2022; Blessing et al., 2017; Kurganovs and Engedal, 2024). AR mutations (e.g., ligand-binding domain mutations in LNCaP cells) further disrupt this network, leading to aberrant responses to anti-androgens such as bicalutamide (Tran et al., 2009; Taplin and Balk, 2004). Overall, this crosstalk forms a complex regulatory network that underlies the transition from hormone-dependent to -independent prostate cancer (Takayama and Inoue, 2013).

### 5.2 From intracellular homeostasis to tissue microenvironment: senescence-associated secretory phenotype (SASP)

Proteostasis imbalance in senescent cells—such as accumulation of misfolded proteins, endoplasmic reticulum stress, and advanced glycation end products (AGEs)—activates the unfolded protein response (UPR) and NF-κB pathways, driving SASP production and secretion, thereby remodeling the prostate tissue microenvironment and creating an inflammatory “soil” for tumor initiation (López-Otín et al., 2013; Melia et al., 2022). SASP comprises a spectrum of pro-inflammatory cytokines and growth factors, including IL-6, IL-8, CXCL1, and GM-CSF, which are amplified in therapy-induced senescence (e.g., chemotherapy, radiotherapy, or anti-androgen treatment) (Melia et al., 2022; Krtolica et al., 2001). Proteostasis collapse activates UPR, promoting transcription and secretion of these factors, generating a positive feedback loop (Melia et al., 2022; Coppé et al., 2010). Specifically, proteostasis collapse in aging stromal fibroblasts triggers constitutive NF-κB signaling, leading to the sustained release of IL-6 and GROα, which paracrinely stimulate tumor proliferation and invasion.

SASP remodels the tumor microenvironment *via* paracrine effects: it promotes tumor growth and immune suppression, e.g., CXCL1 and CXCL2 recruit myeloid-derived suppressor cells (MDSCs), suppressing CD8^+^ T cell and NK cell antitumor activity and facilitating immune evasion (Melia et al., 2022; Krtolica et al., 2001); it enhances malignant phenotypes, as IL-6 and IL-8 induce epithelial-mesenchymal transition (EMT) and invasiveness, promoting tumor progression (Melia et al., 2022; Krtolica et al., 2001; Coppé et al., 2010); and it drives metabolic reprogramming, with senescence-associated lipid dysregulation (e.g., sphingolipid accumulation) amplifying inflammatory signaling (Li and Kim, 2021). AR signaling and SASP exhibit bidirectional regulation: AR activation suppresses certain SASP factors, whereas anti-androgen therapy-induced senescence (e.g., bicalutamide or enzalutamide) enhances SASP’s pro-tumor effects, exacerbated in elderly or diabetic contexts (Melia et al., 2022; Taplin and Balk, 2004). Single-cell omics analyses reveal that microenvironmental heterogeneity further promotes clonal evolution in prostate cancer (Yu et al., 2023). Epidemiologically, this mechanism contributes to age-related increases in prostate cancer incidence (López-Otín et al., 2013).

**FIGURE 1:**
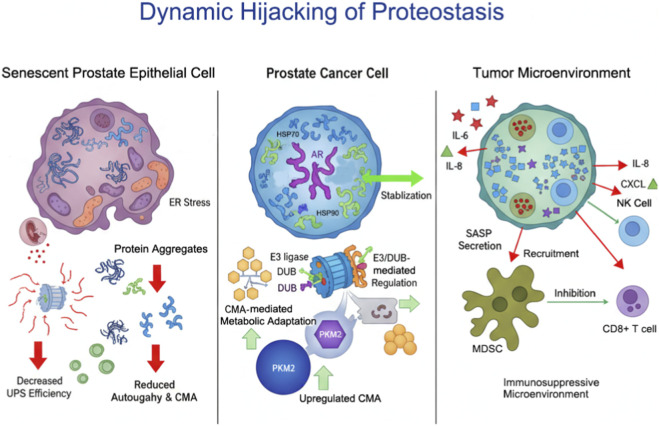
Integrated Mechanisms of Proteostasis Network Dysregulation in Prostate Cancer. This schematic illustrates the interplay between aging-associated proteostasis network (PN) dysregulation and prostate cancer (PCa) progression. Aging induces functional decline in molecular chaperones, the ubiquitin-proteasome system (UPS), and autophagy pathways, leading to protein misfolding, ER stress, and mitochondrial dysfunction. Tumor cells hijack these dysregulated PN components to stabilize androgen receptor (AR) signaling *via* HSP90/HSP70, modulate protein fate through E3 ligases and deubiquitinases (DUBs), and fine-tune metabolism *via* chaperone-mediated autophagy (CMA). The resulting cellular changes drive proliferation and survival, while senescence-associated secretory phenotype (SASP) factors remodel the tumor microenvironment, recruit myeloid-derived suppressor cells (MDSCs), and suppress CD8^+^ T and NK cell activity, thereby promoting immune evasion and tumor malignancy.

Proteostasis imbalance drives intrinsic proliferation and survival of prostate cancer cells *via* AR-centric network dysregulation, while SASP-mediated microenvironment remodeling promotes immunosuppression and malignant evolution. Targeting these cooperative mechanisms—such as HSP90 inhibition, autophagy modulation, or SASP neutralization—offers promising therapeutic avenues for prostate cancer. The integrated interplay among chaperones, UPS, and autophagy pathways, and their impact on AR signaling and the tumor microenvironment, is summarized in Figure 1.

## 6 Therapeutic prospects and challenges

The imbalance of the proteostasis network in prostate cancer offers multidimensional therapeutic opportunities, including modulation of molecular chaperones, the ubiquitin-proteasome system (UPS), and autophagy pathways. However, current strategies—such as HSP90 inhibitors, proteasome inhibitors, and autophagy inhibitors—face significant clinical challenges, including toxicity, resistance, and limited efficacy (Kumar et al., 2022; Neckers and Workman, 2012). Future approaches need to shift from broad-spectrum inhibition toward precision targeting, developing agents with higher specificity and leveraging combination strategies to enhance therapeutic outcomes (Andersen et al., 2010; Domingo-Domenech et al., 2005). This section summarizes the current status and limitations of targeting proteostasis and highlights prospects for precision therapies.

### 6.1 Targeting proteostasis: current strategies and limitations

#### 6.1.1 HSP90 inhibitors

HSP90 inhibitors (e.g., ganetespib, onalespib, NVP-AUY922) disrupt HSP90 interactions with the androgen receptor (AR), its variants (e.g., AR-V7), and oncogenic signaling proteins (e.g., HER2, Akt), inducing their degradation and demonstrating anti-tumor potential (Kumar et al., 2022; Neckers and Workman, 2012; Centenera et al., 2012). For instance, ganetespib suppresses AR signaling and induces apoptosis in castration-resistant prostate cancer (CRPC) cell lines (Centenera et al., 2012; Thakur et al., 2016), while NVP-AUY922 exhibits stronger anti-cancer activity than traditional inhibitors (e.g., 17-AAG) *in vitro* and *ex vivo* models (Gandhi et al., 2013). A phase II clinical trial (NCT01485835) evaluated ganetespib combined with docetaxel in CRPC, showing PSA declines in a subset of patients but limited overall survival benefit (Thakur et al., 2016; Slovin et al., 2019).

Despite their mechanistic promise, HSP90 inhibitors are limited by dose-dependent toxicities, including diarrhea, fatigue, and hepatotoxicity. Crucially, a primary reason for the clinical failure of first-generation HSP90 inhibitors was that pan-inhibition led to severe systemic toxicity and compensatory upregulation of HSP70 *via* the heat shock response (HSR), which effectively sustained tumor cell survival despite HSP90 blockade. Moreover, tumors can escape therapy through activation of the heat shock response, such as HSP70 upregulation, or *via* AR splice variants like AR-V7, which are insensitive to HSP90 inhibition. Moreover, single-agent trials have generally failed to achieve significant survival benefits, partly due to poor drug stability and low targeting efficiency (Neckers and Workman, 2012; Slovin et al., 2019).

#### 6.1.2 Proteasome inhibitors

Proteasome inhibitors (e.g., bortezomib) exert anti-tumor effects by inhibiting NF-κB signaling, blocking protein degradation, and inducing apoptosis (Domingo-Domenech et al., 2005; Papandreou et al., 2004). Phase I clinical studies indicate that bortezomib monotherapy in CRPC can reduce PSA levels and IL-6 expression, with partial responses in some patients (Papandreou et al., 2004; Lamprou et al., 2021). Combination therapies (e.g., bortezomib plus docetaxel) demonstrated a 28% PSA response rate in phase II trials but did not significantly improve overall survival (Hainsworth et al., 2007; Tannock et al., 2004).

However, their clinical application is constrained by dose-limiting toxicities, including diarrhea, peripheral neuropathy, and thrombocytopenia. Resistance frequently emerges through activation of alternative pathways, such as Akt upregulation or SRC-3 accumulation, and in some contexts bortezomib may paradoxically promote tumor proliferation. Additionally, CRPC’s lower dependency on UPS contributes to overall modest response rates, limiting its therapeutic impact (Tannock et al., 2004; Morris et al., 2007).

#### 6.1.3 Autophagy inhibitors

Autophagy inhibitors (e.g., chloroquine) block lysosomal acidification and autophagic flux, enhancing the cytotoxicity of chemotherapy or targeted therapy (Lamprou et al., 2021; Parker and Sartor, 2011). Preclinical studies show that chloroquine combined with docetaxel can reverse resistance caused by PLIN3 loss (Lamprou et al., 2021; Parker and Sartor, 2011), and combination with Src kinase inhibitors or enzalutamide suppresses CRPC growth and induces apoptosis (Tran et al., 2009; Wu et al., 2010). Furthermore, triptolide induces protective autophagy *via* CaMKKβ-AMPK activation, highlighting the complexity of autophagy regulation (Ferraldeschi et al., 2016).

The therapeutic application of autophagy inhibitors is hampered by toxicity and lack of tumor selectivity. Long-term chloroquine use can cause retinopathy, myopathy, and immunosuppression, while its broad-spectrum activity may interfere with normal autophagic processes in healthy cells. Moreover, evidence of clinical efficacy remains limited, with most studies conducted in preclinical settings (Ma et al., 2023).

While HSP90 inhibitors, proteasome inhibitors, and autophagy inhibitors are mechanistically feasible, their clinical utility is substantially limited by overlapping challenges, including systemic toxicity, tumor adaptive resistance, lack of specificity, and insufficient therapeutic efficacy. These limitations underscore the urgent need for more precise and targeted strategies (Kumar et al., 2022; Neckers and Workman, 2012; Papandreou et al., 2004).

### 6.2 Future directions: towards “precision targeting”

#### 6.2.1 From broad-spectrum inhibition to precision targeting

To overcome the limitations of broad-spectrum inhibitors, future strategies should focus on developing agents with higher specificity, targeting critical proteostasis nodes in prostate cancer (Andersen et al., 2010).

Small molecule inhibitors targeting prostate cancer-specific E3 ligases (e.g., WWP1, CBL-b, TRAF6) or deubiquitinases (DUBs, e.g., USP14, UCHL5) can reduce off-target effects (Andersen et al., 2010). For example, TRAF6 inhibitors block Akt ubiquitination and activation, suppressing CRPC growth (Matos et al., 2020). DUB inhibitors (e.g., NiPT) induce apoptosis *via* ubiquitination modulation in lung cancer models, indicating potential applicability to prostate cancer, though toxicity optimization is required (Farshi et al., 2015). Small molecule inhibitors targeting prostate cancer-specific E3 ligases (e.g., WWP1, CBL-b, TRAF6) or deubiquitinases (DUBs, e.g., USP14, UCHL5) can reduce off-target effects (Andersen et al., 2010; Grasso et al., 2012). To enhance clinical success rates, future trials should utilize mechanism-based biomarkers, such as tumor LAMP-2A expression levels or AR-V7 status, to identify patient subgroups most likely to respond to specific proteostasis-targeting agents.

Chaperone-mediated autophagy (CMA) supports CRPC survival by regulating AR stability and metabolic reprogramming. Developing specific LAMP-2A inhibitors may overcome the limitations of non-specific autophagy inhibitors like chloroquine (Nguyen et al., 2014; Chen Q. et al., 2021). Preclinical LAMP-2A silencing induces CRPC cell apoptosis, demonstrating therapeutic potential (Tran et al., 2009).

#### 6.2.2 Combination therapy strategies

Combination therapies that simultaneously target proteostasis and existing treatment modalities can enhance efficacy and overcome resistance (Tran et al., 2009; Domingo-Domenech et al., 2005; Slovin et al., 2019).

HSP90 or autophagy inhibitors combined with docetaxel, abiraterone, or enzalutamide synergistically induce cell death and reverse resistance (Tran et al., 2009; Slovin et al., 2019; Valdespino et al., 2007). For instance, onalespib plus abiraterone, although not significantly improving clinical outcomes, illustrates the potential of co-targeting HSP90 and AR pathways (Williams et al., 2003). Bortezomib plus docetaxel induces PSA decline in a subset of patients, highlighting the benefits of combination therapy (Nguyen et al., 2014).

Proteasome inhibitors (e.g., bortezomib) can enhance immune checkpoint inhibitor efficacy (e.g., PD-1/PD-L1 blockade) by modulating the tumor microenvironment, such as reducing MDSC recruitment (Domingo-Domenech et al., 2005). Similarly, autophagy inhibition can improve antigen presentation and potentiate immunotherapy (Kantoff et al., 2010). The clinical success of immunotherapies like sipuleucel-T in CRPC further supports the potential of combination strategies (Kantoff et al., 2010; Devlies et al., 2021). However, targeting proteostasis requires careful optimization, as systemic inhibition (e.g., of the proteasome) may exert a “double-edged” effect: potentially enhancing tumor antigen presentation while simultaneously impairing the viability and function of immune effector cells.

**FIGURE 2:**
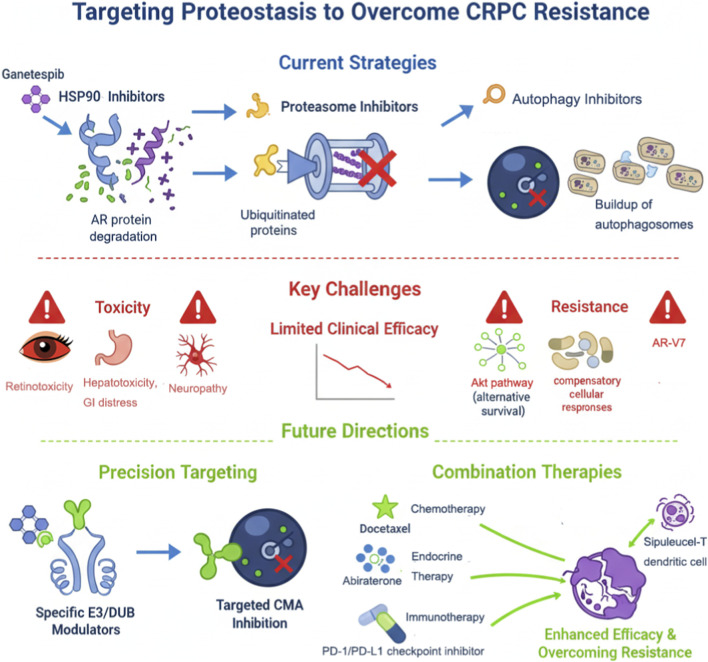
Therapeutic strategies targeting proteostasis in prostate cancer and future directions.

Future treatments should prioritize the development of highly selective agents (e.g., isoform-specific HSP90 inhibitors or E3/DUB modulators), optimize combination regimens to reduce toxicity, and employ biomarkers (e.g., AR-V7 expression, E3/DUB levels, or microenvironment features) to guide personalized therapy (Andersen et al., 2010; Domingo-Domenech et al., 2005; Grasso et al., 2012). Exploration of novel targets such as the CMA pathway and integration with immunotherapy holds promise to overcome current CRPC treatment barriers and achieve substantial survival benefits. Current limitations and future precision-targeting strategies for proteostasis in CRPC are summarized in Figure 2.

This figure depicts current and emerging strategies for targeting proteostasis in prostate cancer. Existing therapies include HSP90 inhibitors, proteasome inhibitors, and autophagy blockers (e.g., chloroquine), which act on AR, misfolded proteins, and CMA substrates. Clinical limitations such as toxicity, resistance mechanisms (AR variants, UPS compensation, autophagy activation), and limited efficacy are indicated. Future directions involve developing highly specific modulators of E3 ligases and DUBs, LAMP-2A/CMA inhibitors, and combination therapies with chemotherapy, endocrine therapy (e.g., abiraterone, enzalutamide), or immunotherapy (e.g., PD-1/PD-L1 inhibitors, Sipuleucel-T) to enhance therapeutic response and overcome CRPC resistance.

## 7 Conclusion and outlook

This review highlights a profound biological paradox linking aging and prostate cancer: the systemic decline of the proteostasis network (PN) associated with aging does not merely cause loss of cellular function but establishes a molecular landscape that can be exploited and remodeled by an age-dependent malignancy. Aging-induced proteostasis imbalance is dynamic and plastic, enabling tumor cells to selectively hijack and amplify specific components that maximize survival advantage. These include reliance on molecular chaperones HSP90/HSP70 to stabilize androgen receptor (AR), manipulation of E3 ligases and deubiquitinases (DUBs) to dictate critical protein fate, and activation of chaperone-mediated autophagy (CMA) to finely regulate metabolism. Consequently, prostate cancer emerges from a transition between widespread, non-specific functional decline in normal aging tissues and a highly specific, pathway-dependent vulnerability in tumor cells, reflecting a co-evolution from “ubiquitous decline” to “targeted dependency.”

Future research should focus on translating these mechanistic insights into clinically actionable strategies. Key directions include developing biomarkers for noninvasive, dynamic assessment of proteostasis network activity in patients, utilizing single-cell and spatial transcriptomic approaches to resolve intratumoral heterogeneity and identify subpopulations with unique proteostasis vulnerabilities, and establishing animal models that more faithfully recapitulate human aging to clarify causal links between proteostasis imbalance and tumor initiation. These approaches will enhance early detection, guide precision targeting of critical proteostasis nodes, and inform combination or preventive therapies in high-risk populations. Ultimately, understanding the dynamic remodeling of proteostasis within the aging context offers novel perspectives on prostate cancer pathogenesis and opens promising avenues for the development of precision interventions against this major age-associated malignancy.
